# The vasoconstrictor adenosine 5′-tetraphosphate is a danger signal that induces IL-1β

**DOI:** 10.1186/s10020-025-01116-6

**Published:** 2025-02-21

**Authors:** Judith Bockstiegel, Jonas Engelhardt, Mirjam Schuchardt, Markus Tölle, Günther Weindl

**Affiliations:** 1 Pharmaceutical Institute, Pharmacology and Toxicology Section, University of Bonn, Gerhard- Domagk-Str. 3, 53121 Bonn, Germany; 2 Department of Nephrology and Medical Intensive Care, Charité-Universitätsmedizin Berlin, Freie Universität Berlin, Humboldt Universität zu Berlin, Hindenburgdamm 30, 12203 Berlin, Germany; 3 Medical School Berlin, Faculty of Medicine, Rüdesheimer Straße 50, 14513 Berlin, Germany

**Keywords:** IL-1beta, Adenosine 5′-tetraphosphate, ATP, NLRP3 inflammasome, Human macrophages, Metallothioneins

## Abstract

**Background:**

The endogenous nucleotide adenosine 5′-tetraphosphate (Ap4) is a potent vasoconstrictor. Despite its structural similarity to the danger signal adenosine 5’-triphosphate (ATP), the immunomodulatory effects of Ap4 remain unclear.

**Methods:**

Modulation of interleukin (IL)-1β secretion by Ap4 was studied in both immune cells lines (THP-1, U937) and primary immune cells. Genetic and pharmacological approaches were used to characterize signaling. Cytokine production was measured using ELISA and multiplex assays, while cell viability was determined by MTT and LDH assays. Calcium influx and YO-PRO-1 uptake were assessed via microplate assays and flow cytometry, respectively. RNA sequencing and Western blotting were performed to analyze global gene expression and protein levels.

**Results:**

We demonstrate that Ap4 stimulates IL-1β release in primed immune cells without affecting the levels of other cytokines, suggesting specificity in its immunomodulatory actions. Mechanistically, Ap4-induced IL-1β release was partially modulated by the P2X7 receptor, a key mediator of inflammation. However, unlike canonical inflammasome activators, this process was independent of potassium efflux, the NLRP3 inflammasome, and caspase-1. Ap4 specifically increased LDH release in macrophages irrespective of priming. Furthermore, Ap4-mediated calcium influx, crucial for immune cell activation, predominantly occurred through P2Y receptors rather than P2X7 receptors. Transcriptomic analysis highlighted Ap4-induced upregulation of metallothioneins, implicating metal ion homeostasis in Ap4-mediated responses.

**Conclusions:**

Collectively, our findings suggest Ap4 as a novel pro-inflammatory mediator capable of inducing IL-1β release in innate immune cells through distinct mechanisms from classical NLRP3 inflammasome activators, shedding light on its potential role in inflammatory diseases and vascular disorders.

**Supplementary Information:**

The online version contains supplementary material available at 10.1186/s10020-025-01116-6.

## Background

Adenosine 5’-tetraphosphate (Ap4) is an endogenous nucleotide composed of an adenosine molecule linked to a linear chain of four phosphate groups via its ribose 5’ end. Ap4 structurally resembles adenosine triphosphate (ATP) but contains an additional phosphate group. Originally discovered in mammalian striated muscle, Ap4 has since been detected in various tissues, including platelets and human plasma (Pietrowska-Borek et al. 2020; Tölle et al. 2008). Plasma concentrations are twice as high as those of ATP (Tölle et al. 2008). While endogenous biosynthesis and metabolism of Ap4 is primarily enzymatic, their mechanisms remain incompletely understood (Amici et al. 2017). Ap4 degrades into ATP, however, Ap4 has much greater stability compared to ATP (Mateo et al. 1997; Gómez-Villafuertes et al. 2000). After two minutes, Ap4 undergoes only a 2% hydrolysis rate, while ATP demonstrates a markedly higher rate of 75% hydrolysis (Gómez-Villafuertes et al. 2000). The physiological functions of Ap4 are yet to be fully elucidated. It is believed that Ap4 is released from platelets or chromaffin granules into the bloodstream, where it inhibits thrombin-mediated platelet aggregation or regulates blood pressure by activating both metabotropic (P2Y) and ionotropic (P2X) purine receptors (Gómez-Villafuertes et al. 2000). Importantly, Ap4 acts as a potent and long lasting endothelium-derived vasoconstrictor in the kidney by stimulating P2X1 receptors (Tölle et al. 2008).

P2Y receptors, a subset of G-protein-coupled receptors, are activated by adenine or uridine nucleotides and have been implicated in immune regulation and inflammation (Von Kügelgen and Hoffmann 2016; Schuchardt et al. 2012). They are divided into G_q_- (P2Y1, P2Y2, P2Y4, P2Y6, P2Y11), which upon activation increase intracellular calcium through phospholipase C and inositol 1,4,5-trisphosphate (IP_3_), releasing calcium from intracellular stores, and G_i_-coupled (P2Y12, P2Y13, P2Y14) receptors. P2X receptors are ubiquitously expressed in vertebrates and modulate various biological processes (North 2002; Di Virgilio et al. 2017). Comprising seven genes (P2X1-P2X7), they form ion channels as homo- or heteromers with two transmembrane domains and a large extracellular loop. Ligand binding, such as ATP, induces conformational changes, permitting cation influx (sodium and calcium) and efflux (potassium). The P2X7 receptor is primarily involved in inflammatory responses and is expressed on immune cells such as macrophages (Di Virgilio et al. 2017). In healthy tissue, P2X7 receptors remain inactive due to their low affinity for ATP. However, elevated ATP levels at sites of inflammation activate these receptors, subsequently initiating nucleotide-binding oligomerization domain, leucine-rich repeat receptor, and pyrin-domain containing-protein 3 (NLRP3) inflammasome activation. The NLRP3 inflammasome, a cytoplasmic multiprotein complex, is generally activated in two steps (Swanson et al. 2019; Fu and Wu 2023). First, priming by Toll-like receptors (TLRs) induces NF-κB mediated expression of pro-interleukin (IL)-1β and NLRP3-associated proteins. Second, NLRP3 inflammasome assembly occurs, resulting in activation of caspase-1, which cleaves pro-IL-1β into IL-1β, and leads to cell death by pyroptosis. Extracellular release of IL-1β is triggered by potassium efflux mediated by P2X7 receptor stimulation or pore-forming substances such as nigericin. However, we and others have also reported mechanisms for IL-1β release that are independent of NLRP3 (Mizushina et al. 2019; Bockstiegel et al. 2023).

The NLRP3 inflammasome is linked with persistent, low-grade, sterile inflammation observed during physiological ageing, known as “inflammaging” (Franceschi et al. 2000; Latz and Duewell (2018); Herrmann et al. 2021), which exacerbates the development and progression of renal failure (Mihai et al. 2018). Furthermore, IL-1β-related inflammatory responses can accelerate vascular calcification, a critical factor in the pathophysiology of cardiovascular disease (Herrmann et al. 2021; Wen et al. 2013; Ridker et al. 2017). Vascular wall calcification and chronic inflammation are prevalent in chronic kidney disease, correlating with increased morbidity and mortality rates (Vanholder et al. 2005; Mizobuchi et al. (2009); Tölle et al. (2015); Lanzer et al. 2021).

Although Ap4 shares structural resemblance with ATP, immunomodulatory effects have not yet been elucidated. Ap4 activates several P2 receptors, including P2Y1 (Gómez-Villafuertes et al. 2000), P2X1 (Tölle et al. 2008), and P2X4 (Jones et al. 2000), therefore we hypothesized its potential to stimulate P2X7 receptors and inducing NLRP3-dependent IL-1β release in immune cells. Considering that P2X7 receptors are expressed in the kidney and upregulated in pathological conditions like chronic renal failure and inflammatory kidney diseases (Menzies et al. 2017), the activation of these receptors by Ap4 might contribute to the pathophysiology of vascular diseases and chronic kidney conditions.

Here, we uncover a previously unrecognized pro-inflammatory role of Ap4 in stimulating the release of bioactive IL-1β in immune cells. Mechanistically, we report that IL-1β release is partially dependent on P2X7 receptor activity but occurs independently of K^+^ efflux, NLRP3 inflammasome and caspase-1 activation. This is supported by both pharmacological and genetic approaches and indicates distinct mechanisms of action for ATP and Ap4. Thus, our findings highlight the potent vasoconstrictor Ap4 as a novel danger signal to induce IL-1β release.

## Methods

### Cell culture

THP-1 cells (ACC 16, DSMZ-German Collection of Microorganisms and Cell Cultures GmbH, Braunschweig, Germany), U937 cells (300368, Cell Lines Service GmbH, Eppelheim, Germany) or NLRP3-KO THP-1 cells (thp-konlrp3z, Invivogen, Toulouse, France) were cultured in growth medium consisting of RPMI 1640 (11530586, Fisher scientific, Schwerte, Germany) supplemented with 100 U/ml penicillin and 100 µg/ml streptomycin (P4333, Sigma-Aldrich, Taufkirchen, Germany), 2 mM l-glutamine (G7513, Sigma-Aldrich, Taufkirchen, Germany) and 10% heat-inactivated fetal bovine serum (FBS; S0615, Sigma-Aldrich, Taufkirchen, Germany) at a density of 2–8 × 10^5^ cells/ml. THP-1 cells were used from passages 4 to 25, and U937 cells from passages 21 to 38. Both cell lines were maintained at 37 °C in a humidified atmosphere of 5% CO_2_ and 95% air. Cell lines were regularly tested negative for mycoplasma contamination (VenorGeM Classic Mycoplasma PCR detection kit, 11-8100, Minerva Biolabs, Berlin, Germany). To generate THP-1-derived macrophages, THP-1 monocytes were seeded into 24-well plates at a density of 4 × 10^5^ cells/ml in growth medium including 25 ng/ml PMA (phorbol 12-myristate 13-acetate; tlrl-pma, Invivogen, Toulouse, France). After 48 h, adherent cells were carefully washed with PBS (phosphate buffered saline; P04-53500, Pan Biotechne, Aidenbach, Germany) and rested in PMA-free medium for 24 h.

For generating U937 macrophages, U937 cells were seeded into 24-well plates at a density of 2 × 10^5^ cells/ml in growth medium including 50 ng/ml PMA. After 72 h, adherent cells were carefully washed with PBS and rested in PMA-free medium for 24 h. U937 monocytes and macrophages were characterized by expression of differentiation markers and their ability to respond to lipopolysaccharide (LPS) (Fig. S1). Fc receptors of harvested cells were blocked using Human TruStain FcX (#422302, BioLegend, London, UK) and labelled with the following fluorophore-conjugated mouse (all IgG1, κ) monoclonal antibodies: BV421-conjugated-anti-CD11b (clone ICRF44; #301323, BioLegend, London, UK), PE-conjugated anti-CD11c (clone 3.9; #301605, BioLegend, London, UK), APC/Fire750 conjugated-anti-CD14 (clone.63D3; #367119, BioLegend, London, UK) and AlexaFluor647-conjugated-anti-CD86 (clone IT2.2; #305415, BioLegend, London, UK) according to the manufacturers’ protocols. Cells were analyzed by flow cytometry (CytoFlex flow cytometer, Beckman Coulter, Krefeld, Germany). U937 monocytes and macrophages were incubated with LPS from *Escherichia coli* 0111:B4 (tlrl-3pelps, Invivogen, Toulouse, France) for 4 h and cell culture supernatants were assayed for tumor necrosis factor (TNF) by commercially available enzyme-linked immunosorbent assay (ELISA) (88-7346-88, Thermo Fisher Scientific, Darmstadt, Germany).

Peripheral blood mononuclear cells (PBMCs) were isolated from buffy-coat donations (Institute of Experimental Haematology and Transfusion Medicine, University Clinic Bonn) by density gradient centrifugation as described previously (Bockstiegel et al. 2023; Engelhardt et al. 2024) using Bicoll separation media (BS L6115, Bio&Sell, Nuremberg, Germany).

HEK293 cells (ACC305, DSMZ-German Collection of Microorganisms and Cell Cultures GmbH, Braunschweig, Germany) were cultured in Dulbecco’s modified Eagle’s medium (DMEM; P04-03500, Pan Biotechne, Aidenbach, Germany) containing 4.5 g/l glucose, 10% (v/v) heat-inactivated FBS (S0615, Sigma-Aldrich, Taufkirchen, Germany), 2 mM l-glutamine (G7513, Sigma-Aldrich, Taufkirchen, Germany).

HEK-blue IL-1β cells were cultured in DMEM (P04-03500, Pan Biotechne, Aidenbach, Germany) containing 4.5 g/l glucose, 10% (v/v) heat-inactivated FBS (S0615, Sigma-Aldrich, Taufkirchen, Germany), 2 mM l-glutamine (G7513, Sigma-Aldrich, Taufkirchen, Germany), 100 U/ml penicillin and 100 µg/ml streptomycin (P4333, Sigma-Aldrich, Taufkirchen, Germany), and the selection antibiotics 100 µg/ml normocin (ant-nr-05, Invivogen, Tolouse, France), 100 µg/ml zeocin (ant-zn-05, Invivogen, Tolouse, France). Cells were used from passage 3 to 20 and maintained at 37 °C in a humidified atmosphere of 5% 5CO_2_ and 95% air. To determine if THP-1 stimulated cells secrete bioactive form of IL-1β, a total of 50 µl of THP-1 supernatant was transferred to a 96-well tissue culture–treated plate and mixed with 5 × 10^4^ IL-1β reporter cells in test media (culture media without normocin and zeocin) and incubated for 20 h at 37 °C. After IL-1β receptor stimulation of HEK-Blue cells, NF-κB and AP-1 activation induced production of secreted embryonic alkaline phosphatase (SEAP) that can be determined with the colorimetric substrate QuantiBlue (rep-qbs, Invivogen, Toulouse, France) by reading the optical density (OD) at 620 nm.

### Cell stimulation

PBMCs as well as THP-1 monocytes and differentiated THP-1 derived macrophages were primed with Pam_3_CSK_4_ (1 µg/ml; tlrl-pms-1, Invivogen, Toulouse, France) or growth medium for 3 h. Afterwards PBMCs and THP-1 macrophages were stimulated for additional 3 h or THP-1 monocytes for 6 h with Ap4 (if not otherwise specified 5 mM; NU-1102-CSTM, Jena Bioscience, Jena, Germany) or ATP (if not otherwise specified 5 mM; NU-1010-100G, Jena Bioscience, Jena, Germany). In selected experiments, cells were preincubated with the non-competitive P2X7 receptor antagonist A804598 (1 µM; 4473, Tocris Bioscience, Bristol United Kingdom), irreversible P2X7 receptor antagonist oxidized ATP (oxATP) (300 µM; 505758, Merck, Darmstadt, Germany), P2X4 receptor antagonist 5-BDBD [5-(3-Bromophenyl)-1,3-dihydro-2 H-Benzofuro[3,2-e]-1,4-diazepin-2-one] (25 µM; SML0450-5MG, Sigma-Aldrich, Taufkirchen, Germany), P2 receptor antagonist PPADS (100 µM; 0625, Tocris Bioscience, Bristol, United Kingdom), G_q/11_ inhibitor YM-254,890 (1 µM; AG-CN2-0509, Biomol, Hamburg, Germany), NLRP3 inhibitor MCC950 (10 µM; 5479, Tocris Bioscience, Bristol, United Kingdom) or NF-κB inhibitor Bay 11-7082 (20 µM; B5556, Sigma-Aldrich, Taufkirchen, Germany), Caspase-1 inhibitor Ac-YVAD-cmk (40 µM; 10014, Biomol, Hamburg, Germany), pan-caspase inhibitor Z-VAD-fmk (40 µM; tlrl-vad, Invivogen, Toulouse, France), serine protease inhibitor AEBSF (300 µM; 50985.100, Biomol, Hamburg, Germany), cysteine protease inhibitor E64 (10 µM; 324890, Merck, Darmstadt, Germany), aspartic protease inhibitor pepstatin A (50 µM; 2936, Carl Roth, Karlsruhe, Germany), l-homocysteine (100 µM; Cay30852-5, Biomol, Hamburg, Germany), or ion chelator TPEN [N, N,N′,N′-tetrakis(2-pyridinylmethyl)-1,2-ethanediamine] (10 µM; Cay13340-10, Biomol, Hamburg, Germany) for 1 h before stimulation. To determine TNF or IL-8 release, THP-1 macrophages were incubated with Ap4 (5 mM) or Pam_3_CSK_4_ (1 µg/ml) for 4 or 24 h, respectively. To determine the contribution of the NLRP3 inflammasome, NLRP3-KO THP1 macrophages were primed with Pam_3_CSK_4_ (1 µg/ml) for 3 h followed by stimulation with Ap4 for 3 h.

### Transfection of HEK293 cells

HEK293 cells were transfected with pUNO1-hP2RX7 (puno1-hp2rx7) or pUNO1-mcs (puno1-mcs, both Invivogen, Toulouse, France) as described (Pfalzgraff et al. 2018). Briefly, cells (0.625 × 10^5^) were cultured in poly-L-lysine (P6282-5MG, Sigma-Aldrich, Taufkirchen, Germany)-coated 6-well plates in DMEM (P04-03500, Pan Biotechne, Aidenbach, Germany) containing 4.5 g/l glucose, 5% (v/v) heat-inactivated FBS (S0615, Sigma-Aldrich, Taufkirchen, Germany), 2 mM l-glutamine (G7513, Sigma-Aldrich, Taufkirchen, Germany) at 37 °C and 5% CO_2_. After 24 h, cells were incubated for 4 h at 37 °C and 5% CO_2_ in transfection medium. Polyethylenimine (PEI) MAX (24765, Polysciences Europe, Hirschberg an der Bergstrasse, Germany) was then added at a 1:4 plasmid/PEI (w:w) ratio containing 3 µg plasmid per well. After 48 h at 37 °C and 5% CO_2_, medium was replaced with fresh transfection medium, and the plate was incubated for another 24 h. P2X7 receptor expression was verified by western blot.

### YO-PRO-1 uptake

Transfected HEK293 cells (0.4 × 10^6^ cells/well) were incubated for 60 min at 37 °C with BzATP (300 µM) or Ap4 (5 mM) and YO-PRO-1 (oxazole yellow, 2 µM; HB6210, Hello bio, Princeton, United States) in YO-PRO-assay buffer (pH 7.4) containing HEPES (4-(2-hydroxyethyl)-1-piperazineethanesulfonic acid) (10 mM; RDD002, Sigma-Aldrich, Taufkirchen, Germany), glucose (13 mM; X997.1, Carl Roth GmbH, Karlsruhe, Germany), potassium chloride (2 mM; P017.1, Carl Roth GmbH, Karlsruhe, Germany), calcium chloride (0.1 mM; 793639, Sigma-Aldrich, Taufkirchen, Germany) in demineralized water. Heat-killed cells (70 °C for 10 min) served as a positive control. Afterwards, cells were washed twice with YO-PRO-assay buffer. YO-PRO-1 uptake was determined by flow cytometry analysis (CytoFlex flow cytometer, Beckman Coulter, Krefeld, Germany) in the FITC channel, evaluating YO-PRO-1 positive cells in comparison to the unstimulated sample stained with YO-PRO-1.

### Calcium influx assay

THP-1 monocytes, THP-1 macrophages or transfected HEK293 cells were cultured at a density of 0.1 × 10^6^ cells/well on a poly-l-lysine (P6282-5MG, Sigma-Aldrich, Taufkirchen, Germany)-coated 96-well plate (Corning black, 7341609, VWR, Germany). Cells were incubated for 1 h at room temperature with Hank’s Balanced Salt Solution (HBSS; 14025092, Thermo Fisher scientific, Darmstadt, Germany) containing HEPES (20 mM; RDD002, Sigma-Aldrich, Taufkirchen, Germany), probenecid (1 mM; P8761-25G, Sigma-Aldrich, Taufkirchen, Germany), Fluo4-AM (3 µM; 20552, AAT Bioquest, Pleasanton, United States) and pluronic acid (0.02%; 20052, AAT Bioquest, Pleasanton, United States). Afterwards, the supernatant was replaced with wash buffer (HBSS containing HEPES and probenecid) which may also contain the antagonist PPADS (100 µM; 0625, Tocris Bioscience, Bristol, United Kingdom) or the inhibitor YM-254,890 (1 µM; AG-CN2-0509, Biomol, Hamburg, Germany). If antagonists or inhibitors were present, the plate was incubated for 30 min in the absence of light. Fluorescence (absorbance: 490 nm, emission: 525 nm, cutoff: 515 nm) was recorded by an automatic fluorescence plate reader (Flexstation 3, Molecular Devices, San José, United States) for 90 s at 1.5 s intervals. The fluorescence intensity was expressed as relative fluorescence unit (RFU). During the measurement, ATP (final concentration: 100 µM), Ap4 (0.01 to 100 µM) or the positive control calcimycin, also known as A23187 (10 µM; C7522, Sigma-Aldrich, Taufkirchen, Germany), was automatically injected into the wells after 20 s. RFU values were normalized to the mean values of basal level.

### ELISA

After 3 h of stimulation of Pam_3_CSK_4_-primed PBMCs or THP-1 macrophages or after 6 h of stimulation of Pam_3_CSK_4_-primed THP-1 monocytes cell culture supernatants were collected and analyzed for IL-1β secretion using a commercially available ELISA kit (88-7261-88, Thermo Fisher Scientific, Darmstadt, Germany). After 4 or 24 h of stimulation of THP-1 macrophages cell supernatants were collected and analyzed for TNF or IL-8, respectively, using ELISA kits for TNF (88-7346-88, Thermo Fisher Scientific, Darmstadt, Germany) or IL-8 (88-7066-88, Thermo Fisher Scientific, Darmstadt, Germany).

### Cytokine multiplex-assay

After stimulation of PBMCs cell culture supernatants were collected and centrifuged at 200×* g* for 10 min at 4 °C generate a cell-free preparation. Cytokine concentrations (GM-CSF, IFN-α, IFN-γ, IL-2, IL-4, IL-5, IL-6, IL-9, IL-10, IL-12p70, IL-17 A and TNF) in the supernatants were measured with flow cytometry (CytoFlex flow cytometer, Beckman Coulter, Krefeld, Germany) using MACSPlex Cytokine 12 Kit (130-099-169, Milteny Biotec, Bergisch Gladbach, Germany) according to the manufacturer´s protocol.

### Lactate dehydrogenase (LDH) assay

LDH assay was performed according to the manufacturer’s instructions (CyQUANT; C20301, Thermo Fisher Scientific, Darmstadt, Germany). The percentage of LDH release was calculated compared to 100% cell lysis control.

### MTT assay

Effects on cell viability were analyzed by MTT test in U937 macrophages. U937-differentiated macrophages were incubated with MCC950 (40 µM) for 3 h. Dimethyl sulfoxide (DMSO) (10%, v/v) served as cytotoxic positive control. Afterwards, MTT (3-(4,5-dimethylthiazol-2-yl)-2,5-diphenyltetrazolium bromide, M6494, Thermo Fisher scientific, Darmstadt, Germany) solution (final concentration 1 mg/ml) was added for 4 h. After removing supernatants and solubilization of formazan crystals in DMSO, absorption was determined at 540/10 BP (Mithras2 LB 943, Berthold Technologies, Bad Wildbad, Germany). The viability of unstimulated cells was defined as 100%.

### RNA sequencing and data analysis

Total RNA isolation was performed using innuPREP RNA Mini Kit 2.0 (845-KS-2040050, AnalytikJena, Jena, Germany) according to the manufacturer’s protocol. The library preparation and sequencing were conducted by NGS Core Facility at the University of Bonn, Germany. Library prep was performed using the Lexogen QuantSeq 3′ mRNA-Seq Library Prep Kit FWD and 200 ng RNA as input. Sequencing was done with 1 × 100 bp on NovaSeq 6000 S1 100 Flow cell and with 10 M reads per sample. Three biological replicates for each treatment were used.

The analysis was performed by the Core Unit for Bioinformatics Data Analysis, University of Bonn. After trimming of the Illumina Universal Adapter with cutadapt (Martin 2011) reads have been aligned to the human genome (GRCh38) with STAR (Dobin et al. 2013). FeatureCounts (Liao et al. 2014) was used to assign reads to genes as defined by Ensembl. A read is counted, firstly, if it is uniquely mapped, secondly, if it matches the strand of the gene, and thirdly, if it overlaps with only one gene, i.e. the read can be non-ambiguously assigned to a single gene. Only genes with at least 2 samples each with a minimum count of 50 were considered in the downstream statistical analysis which was performed in the R environment (R 2023) with the Bioconductor package DESeq2 (Love et al. 2014; Huber et al. 2015). Statistical contrasts were calculated for each pair of groups to determine differentially expressed genes. The Benjamini-Hochberg method was used to calculate multiple testing adjusted p-values (false discovery rate, FDR) for each contrast. Data visualizations, such as volcano plots and heatmaps were generated using R-packages ggplot2 (Wickham 2009) and ComplexHeatmap (Gu et al. 2016), respectively. Pathway enrichment analysis for differently expressed genes (FDR < 0.05, Fisher test) was performed using the Bioconductor package clusterProfiler (Wu et al. 2021). Only pathways with a set size of at least 10 or at most 500 genes were considered.

### Western blot

Western blot analysis was performed as previously described (Bockstiegel et al. 2023). For analyzing protein amount in supernatants, THP-1 cells were stimulated in serum free media. Following stimulation, supernatants were collected and centrifuged at 200×*g* for 5 min at 4 °C. Before western blot analysis, proteins in supernatants were concentrated by centrifugation at 14,000×*g* for 20 min to 40 min at 4 °C through a spin filter with a cut-off of 10 kDa (Microcon, MRCPRT010, Merck, Darmstadt). THP-1 cells were washed with ice-cold PBS and harvested in RIPA buffer with protease/phosphatase inhibitor cocktail (5872, Cell Signaling Technology, Leiden, The Netherlands). Lysates were centrifuged at 14,000×*g* for 30 min at 4 °C. The protein amount was determined by bicinchoninic acid assay (Pierce BCA Protein Assay Kit; 23227, Thermo Fisher Scientific, Darmstadt, Germany). Samples containing 20 µg protein were boiled in SDS-Page sample buffer (Lämmli sample buffer, 1610747, BioRad, Feldkirchen, Germany) in the presence of DTT (DL-Dithiothreitol; D-0632, Sigma-Aldrich, Taufkirchen, Germany) and separated on a 10% TGX StainFree FastCast acrylamide gel (1610183, Bio-Rad, Feldkirchen, Germany) containing TEMED (2367, Carl Roth, Karlsruhe, Germany) and ammonium persulfate (A3678, Sigma-Aldrich, Taufkirchen, Germany) using the MiniPROTEAN electrophoresis system (Bio-Rad, Feldkirchen, Germany). To determine total amount of proteins in the membranes, gels were activated using the StainFree gel setting of the ChemiDoc Imager before blotting. Afterwards, gels were blotted on low-fluorescence polyvinylidene difluoride (PVDF) membranes (Immobilon-FL 0,45 μm pore size; IPFL00005; for caspase-1 detection in supernatant-samples: 0.2 µM pore size, 22860, Thermo Fisher scientific, Darmstadt, Germany) using the Trans-Blot Turbo Transfer System (Bio-Rad, Feldkirchen, Germany). After blotting, membranes were analyzed for the total amount of protein followed by membrane blocking with 5% skimmed milk (T145.3, Carl Roth GmbH, Karlsruhe, Germany) in TBS-T buffer consisting of TRIS HCl (T3253, Sigma-Aldrich, Taufkirchen, Germany), NaCl (27810.295, VWR, Darmstadt, Germany) and Tween 20 (9127.1, Carl Roth GmbH, Karlsruhe, Germany) for 1 h at room temperature. Afterwards, membranes were incubated with anti-pro-caspase-1 rabbit mAb (1:1000; #3866, Cell Signaling Technology, Leiden, The Netherlands), anti-pro-IL-1β rabbit mAb (1:1000; #12703, Cell Signaling Technology, Leiden, The Netherlands), anti-cleaved caspase-1 Rabbit mAb (1:1000; #4199, Cell Signaling Technology, Leiden, The Netherlands), anti-hP2X7R rabbit mAb (1:1000; #13809, Cell Signaling Technology, Leiden, The Netherlands), anti-NLRP3 rabbit mAB (1:1000, #13158, Cell Signaling Technology, Leiden, The Netherlands) over night at 4 °C. Anti-rabbit HRP conjugated antibody (1:2000; #7074, Cell Signaling Technology, Leiden, The Netherlands) was incubated for 1 h at room temperature. Blots were developed with ECL reagent (Clarity Western ECL Substrate; 1705060, Bio-Rad, Feldkirchen, Germany) and imaged using ChemiDoc imaging system (Bio-Rad, Feldkirchen, Germany). Values of protein expression were analyzed by densiometry and normalized to total protein levels using Image lab 6.1 (Bio-Rad, Feldkirchen, Germany). Uncropped western blots are shown in Fig. S7.

### Statistical analysis

Data are expressed as means ± SEM. For multiple comparisons, statistically significant differences were determined by one-way ANOVA followed by a Dunnett´s or Tukey´s post-test and considered significant at *P ≤ 0.05, **P ≤ 0.01, ***P ≤ 0.001, ****P ≤ 0.0001. For studies of inhibitory effects Ap4- or ATP-induced IL-1β release was set to 100%. All other values were calculated accordingly. Statistical differences were assessed by one-sample *t*-test against 100%. Statistical analysis was performed using GraphPad Prism 8.0 (GraphPad Software Inc., San Diego, CA, USA).

## Results

### Ap4 induces IL-1β release in innate immune cells

**Fig. 1:**
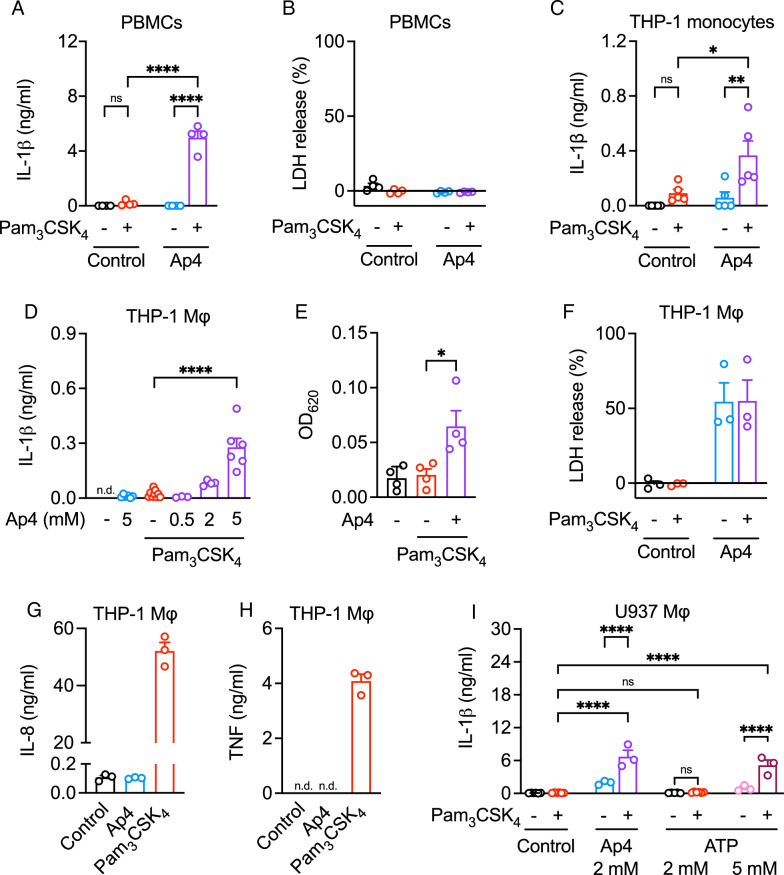
Ap4 induces IL-1β release in immune cells. **A** and **B** PBMCs or **C** THP-1 monocytes were primed without or with Pam_3_CSK_4_ (1 µg/ml) for 3 h and then stimulated with Ap4 (5 mM) for **A** and **B** 3 or **C** 6 h. Cell culture supernatants were analyzed (**A** and **C**) for IL-1β concentration by ELISA and **B** for LDH release. **B** Results are expressed as % of maximum LDH release. Mean ± SEM (*n* = 4–5). One-way ANOVA followed by Dunnett´s post-test, ns ≥ 0.05, *P ≤ 0.05, **P ≤ 0.01, ***P ≤ 0.001, ****P ≤ 0.0001. **D**–**F** THP-1 macrophages were primed with Pam_3_CSK_4_ (1 µg/ml) for 3 h and then stimulated for 3 h with **D** increasing concentrations of Ap4 or **E** and **F** 5 mM Ap4. **D** IL-1β release in cell culture supernatants was determined by ELISA. Mean ± SEM (*n* = 3–6). One-way ANOVA followed by Tukey´s post-test, ****P ≤ 0.0001. **E** Cell culture supernatants of stimulated THP-1 macrophages were transferred to HEK-blue IL-1β reporter cells. SEAP production was detected using QUANTI-Blue and optical density was measured at 620 nm. Mean ± SEM (*n* = 4). One-way ANOVA followed by Dunnett´s post-test, ns ≥ 0.05, *P ≤ 0.05, **P ≤ 0.01, ***P ≤ 0.001, ****P ≤ 0.0001. **F** Cell culture supernatants were analyzed for LDH release. Results are expressed as % of maximum LDH release. Mean + SEM (*n* = 3). **G**, **H** THP-1 macrophages were stimulated **G** 24 h or **H** 4 h with Ap4 (5 mM) or Pam_3_CSK_4_ (1 µg/ml). **G** IL-8 and **H** TNF release in cell culture supernatants was determined by ELISA. Mean ± SEM (*n* = 3). **I** U937 macrophages were primed with Pam_3_CSK_4_ (1 µg/ml) for 3 h and then stimulated with Ap4 (2 mM) or ATP (2 and 5 mM) for 3 h. IL-1β in cell culture supernatants was determined by ELISA. Mean ± SEM (*n* = 3). One-way ANOVA followed by Tukey´s post-test, ns ≥ 0.05, ****P ≤ 0.0001

Extracellular ATP triggers the processing and release of mature IL-1β (Ferrari et al. 1997). We primed human PBMCs with the TLR2/1 ligand Pam_3_CSK_4_, followed by stimulation with 5 mM Ap4, a concentration commonly used for ATP in inflammasome activation. Ap4 induced the release of IL-1β from primed PBMCs (Fig. 1A), although equimolar concentrations of ATP induced 10-fold higher cytokine levels (Bockstiegel et al. 2023). LDH release was not increased by Ap4 (Fig. 1B) which is in contrast to ATP (Bockstiegel et al. 2023). Next, we tested whether Ap4 promotes release of other cytokines besides IL-1β from PBMCs in the presence or absence of Pam_3_CSK_4_. Multiplex analysis showed no increased levels of a panel of cytokines (IL-2, IL-4, IL-5, IL-9, IL-10, IL-12, IL-17 A, GM-CSF, IFN-α, IFN-γ) (Fig. S2). As expected, Pam_3_CSK_4_ induced the release of TNF and IL-6, but again cytokine levels remained unchanged when cells were subsequently stimulated with Ap4.

We and others have shown that the human THP-1 cell line is a suitable model for the investigation of ATP-mediated NLRP3 inflammasome activation and IL-1β release (Bockstiegel et al. 2023; Martinon et al. 2004). In TLR2/1-primed but not unprimed THP-1 monocytes (Fig.1C) and macrophages (Fig. 1D), Ap4 induced a significant and concentration-dependent increase in IL-1β release, although the response was less than in PBMCs. The bioactivity of secreted IL-1β was confirmed using IL-1β reporter cells (Fig. 1E). Stimulation with Ap4 increased LDH levels to a similar extent both in unprimed and primed THP-1 macrophages (Fig. 1F). In line with the results obtained using PBMCs, Ap4 did not increase the levels of IL-8 (Fig. 1G) and TNF (Fig. 1H).

To further characterize Ap4-induced IL-1β release, we used U937 cells which have been also used for inflammasome studies (Song et al. 2017). In contrast to PBMCs, THP-1 monocytes and THP-1 macrophages, priming was dispensable for ATP- and Ap4-induced IL-1β release in U937-derived macrophages, however, TLR2/1-priming further increased IL-1β levels (Fig. 1I). Unexpectedly, Ap4 was more potent than ATP in inducing IL-1β release from U937 macrophages. Ap4, but not ATP, triggered high levels of IL-1β at 2 mM, whereas ATP required 5 mM to induce levels comparable to those induced by 2 mM Ap4.

### Ap4-induced IL-1β release is partially P2X7 receptor-dependent

**Fig. 2:**
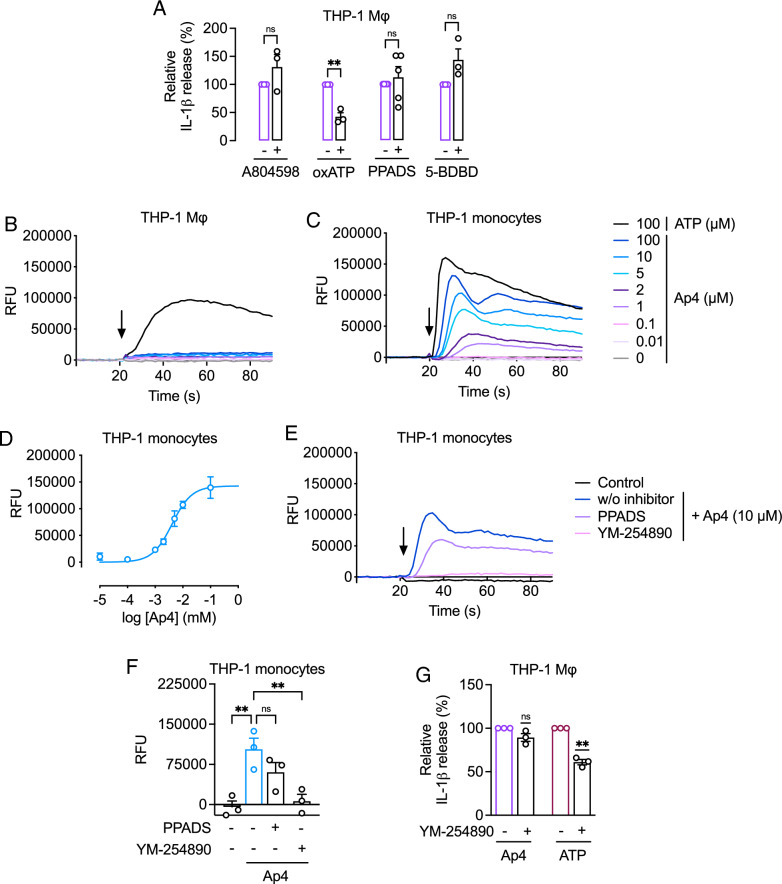
Modulation of Ap4-induced IL-1β release and cytosolic calcium influx by purinergic receptors. **A** THP-1 macrophages were primed with Pam_3_CSK_4_ (1 µg/ml) for 3 h and then stimulated with Ap4 (5 mM) for 3 h. Antagonists of P2X7 receptor (A804598, 1 µM; oxATP, 300 µM), P2X4 receptor (5-BDBD, 25 µM) or P2 receptors (PPADS, 100 µM) were added 1 h before stimulation. IL-1β release in cell culture supernatants was determined by ELISA. Ap4-induced IL-1β release was set to 100%. All other values were calculated accordingly. Mean ± SEM (*n* = 3–5). One-sample *t*-test against 100%, ns ≥ 0.05, **P ≤ 0.01. **B** and **C** HEK293 cells were transfected with the P2X7 receptor (pUNO1-hP2RX7) or the control plasmid (pUNO-mcs). Unprimed (**B**) THP-1 macrophages (**C**) or THP-1 monocytes were loaded with Fluo-4 AM and probenecid. Ap4 at final concentrations of 0.1 to 100 µM or ATP (100 µM) was added after 20 s. Calcium influx was determined by fluorescence intensity measurement. The arrows show the addition of the control or the stimuli. The graph shows the mean values related to the basal level. **B** (*n* = 2), **C** (*n* = 3–5). **D** A concentration-response curve was constructed using the values the maximum RFU. Mean ± SEM (*n* = 3–5). **E** and **F** Unprimed THP-1 monocytes were loaded with Fluo-4 AM and probenecid. Cells were then pre-incubated with P2 receptor antagonist PPADS (100 µM) or G_q/11_ inhibitor YM-254,890 (1 µM) for 30 min. Ap4 (10 µM) was added after 20 s. Calcium influx was determined by fluorescence intensity measurement. The arrow shows the addition of the control or the stimuli. The graph shows the mean values related to the basal level (*n* = 3). **F** A bar chart was created using the respective maximum RFU values of each sample. Mean ± SEM (*n* = 3). One-way ANOVA followed by Dunnett´s post-test, Statistical differences: ns ≥ 0.05, **P ≤ 0.01. **G** THP-1 macrophages were primed with Pam_3_CSK_4_ (1 µg/ml) for 3 h and then stimulated with Ap4 (5 mM) or ATP (5 mM). G_q/11_ inhibitor YM-254,890 (1 µM) was added 1 h before stimulation. IL-1β release in cell culture supernatants was determined by ELISA. IL-1β release induced by Ap4 was set to 100%. All other values were calculated accordingly. Mean ± SEM (*n* = 3). One-sample *t*-test against 100%, ns ≥ 0.05, **P ≤ 0.01

Given its structural similarity to ATP, we hypothesized that Ap4 also triggers IL-1β release through the P2X7 receptor. The non-competitive reversible antagonist A804598 completely blocks IL-1β release by ATP (Bockstiegel et al. 2023), but failed to inhibit IL-1β release induced by Ap4 (Fig. 2A). In the presence of the irreversible P2X7 receptor antagonist oxATP, Ap4-induced IL-1β release was reduced to 43 ± 7% (Fig. 2A) which is in line with ATP (Bockstiegel et al. 2023). Since the P2X4 receptor might be involved in inflammasome activation (De Rivero Vaccari et al. 2012; Gicquel et al. 2015), we tested whether other purine receptors besides the P2X7 receptor are involved in Ap4-mediated IL-1β release. However, neither the P2X4 receptor-specific antagonist 5-BDBD nor the P2 receptor antagonist PPADS decreased IL-1β levels (Fig. 2A). Thus, although the P2X7 receptor appears to be partially involved, IL-1β release from Ap4-stimulated primed macrophages was not entirely prevented by the tested P2X receptor antagonists.

Since extracellular ATP and Ap4 stimulate cytosolic calcium influx through different purinergic receptors (Gómez-Villafuertes et al. 2000; Jones et al. 2000; Vitiello et al. 2012), we compared signaling of ATP and Ap4 in macrophages independent from TLR signaling. As expected, 100 µM ATP induced calcium influx in THP-1 monocytes (Fig. 2B) and macrophages (Fig. 2C) although with different kinetics. In THP-1 macrophages, the initial plateau remained constant during the recording period, whereas a biphasic calcium response was observed in THP-1 monocytes. Ap4 induced a concentration-dependent signal in both THP-1 macrophages and monocytes starting at concentrations of 0.1 and 1 µM, respectively, with an EC_50_ value of 4 µM in THP-1 monocytes (Fig. 2D). However, only a weak response was recorded for Ap4 in THP-1 macrophages. To identify the receptor types that contributed to Ap4-induced calcium influx, we used the P2 receptor antagonist PPADS and the G_q/11_inhibitor YM-254,890 (Takasaki et al. 2004) which also inhibits P2Y receptors coupling to G_q/11_, i.e. P2Y1, P2Y2, P2Y4, P2Y6. In the presence of PPADS a slight but not significant decrease in calcium influx was observed, whereas YM-254,890 completely inhibited Ap4-mediated calcium influx into THP-1 monocytes (Fig. 2E and F). This suggests that Ap4 mediates calcium influx into THP-1 monocytes mainly via P2Y receptors, although we cannot exclude that other GPCRs coupling to G_q/11_may contribute to this effect. Since activation of P2Y receptors has been associated with increased IL-1β release (Zhang et al. 2020), we tested whether Ap4 also mediates IL-1β release via P2Y receptors. In the presence of the G_q/11_ inhibitor YM-254,890, ATP-induced IL-1β release was slightly reduced, but Ap4-induced IL-1β release remained unaffected (Fig. 2G).

**Fig. 3:**
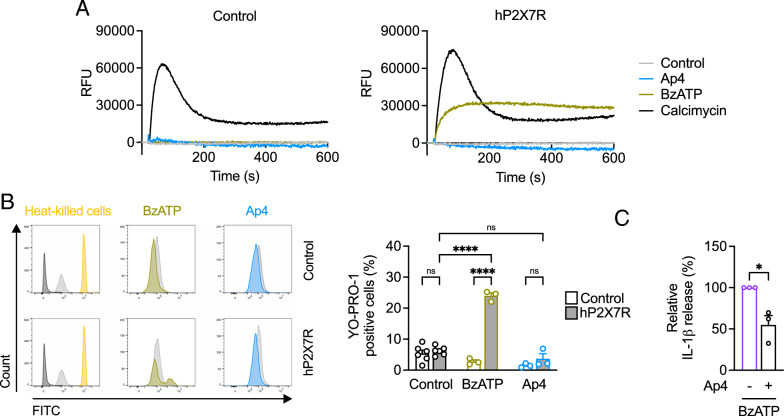
Effects of Ap4 on P2X7 receptor. **A** and **B** HEK293 cells were transfected with P2X7 receptor (pUNO1-hP2RX7) or control plasmid (pUNO-mcs). **A** After transfection, HEK293 cells were loaded with Fluo-4 AM and probenecid. Ap4 (5 mM), BzATP (300 µM) or calcimycin (10 µM) were added after 20 s. Calcium influx was determined by fluorescence intensity measurement. The graph shows the mean values related to the basal level (*n* = 3). **B** Cells were loaded with YO-PRO-1 (2 µM) and stimulated for 1 h without or with BzATP (300 µM) or Ap4 (5 mM). Flow cytometry was used to quantify the change in YO-PRO-1 uptake, as indicated by fluorescence intensity in the FITC channel, after stimulation with either Ap4 or BzATP (depicted by colored curves), in comparison to the untreated control (represented by light grey curves). Heat-killed cells were used as positive control. The dark grey curve shows unstained cells. The histograms are representative of three independent experiments. Mean + SEM (*n* = 3–6). **C** THP-1 macrophages were primed with Pam_3_CSK_4_ (1 µg/ml) for 3 h and then stimulated with BzATP (300 µM) for 3 h. Ap4 (5 mM) was added 1 h before stimulation. IL-1β release in cell culture supernatants was determined by ELISA. IL-1β release induced by BzATP was set to 100%. All other values were calculated accordingly. Bar graphs show mean ± SEM (*n* = 3). One-sample *t*-test against 100%, *P ≤ 0.05

Activation of P2X7 receptors by agonists leads to calcium influx and opening of a membrane pore that is permeable to fluorescent dyes such as YO-PRO-1 (Karasawa et al. 2017). In P2X7 receptor-transfected HEK293 cells (Fig. S3), stimulation with the P2X7 receptor agonist BzATP but not Ap4 resulted in increased calcium influx in cells loaded with the permeable calcium-sensitive fluorescent dye Fluo4-AM (Fig. 3A) as well as increased uptake of YO-PRO-1 compared to control (Fig. 3B). The calcium ionophore calcimycin served as control and induced calcium release into HEK293 cells independent of the P2X7 receptor. Since Ap4-induced IL-1β release could be reduced by the P2X7 receptor antagonist oxATP (Fig. 2A), we hypothesized that Ap4 may interfere with P2X7 receptor activation. In the presence of Ap4, a 55 ± 11% reduction in BzATP-mediated IL-1β cytokine levels was observed (Fig. 3C).

### Ap4-induced IL-1β release in macrophages is independent of K^+^ efflux and NLRP3 inflammasome

**Fig. 4:**
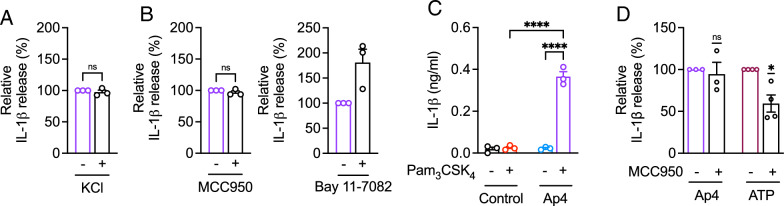
Ap4-induced IL-1β release is potassium- and NLRP3-independent. THP-1 macrophages were primed with Pam_3_CSK_4_ (1 µg/ml) for 3 h and then stimulated with Ap4 (5 mM) **A** in the presence of potassium chloride (75 mM) or **B** without potassium chloride for 3 h. **B** NLRP3 inhibitors MCC950 (10 µM) and Bay 11-7082 (20 µM) were added 1 h before stimulation. IL-1β release in cell culture supernatants was determined by ELISA. IL-1β release induced by Ap4 was set to 100%. All other values were calculated accordingly. Mean ± SEM (*n* = 3). One-sample *t*-test against 100%, ns ≥ 0.05. **C** NLRP3-KO THP-1 macrophages were primed with Pam_3_CSK_4_ (1 µg/ml) for 3 h and then stimulated with Ap4 (5 mM) for 3 h. IL-1β concentration in the supernatants was analyzed by ELISA. Mean ± SEM (*n* = 3). One-way ANOVA followed by Tukey´s post-test, ****P ≤ 0.0001. **D** U937 macrophages were primed with Pam_3_CSK_4_ (1 µg/ml) for 3 h and then stimulated with Ap4 (2 mM) or ATP (5 mM) for 3 h. The NLRP3 inhibitor MCC950 (40 µM) was added 1 h before stimulation. IL-1β release in cell culture supernatants was determined by ELISA. Ap4-induced IL-1β release was set to 100%. All other values were calculated accordingly. Mean ± SEM (*n* = 3–4). One-sample *t*-test against 100%, ns ≥ 0.05

K^+^efflux is crucial for the activation of the NLRP3 inflammasome (Pétrilli et al. 2007). To confirm if Ap4-induced IL-1β release is NLRP3 inflammasome-dependent, we first analyzed whether inhibiting K^+^ efflux inhibits Ap4-induced IL-1β release. While extracellular potassium chloride prevented K^+ ^efflux and nigericin-induced IL-1β release (Bockstiegel et al. 2023), IL-1β release by Ap4 remained unchanged (Fig. 4A). Next, we tested whether Ap4 activates the NLRP3 inflammasome by using pharmacological and genetic inhibition of the NLRP3 inflammasome. In Ap4-stimulated THP-1 macrophages, IL-1β cytokine levels were not decreased in the presence of the NLRP3 inhibitor MCC950 or NF-κB inhibitor Bay 11-7082 (Fig. 4B), indicating that Ap4-induced IL-1β release is NLRP3-independent. This was further supported by a significant increase in IL-1β release in primed NLRP3-KO THP-1 macrophages (Fig. S4) stimulated with Ap4 (Fig. 4C). While ATP-induced IL-1β release in U937 macrophages was reduced by MCC950, Ap4-induced IL-1β remained also unchanged in the presence of MCC950 (Fig. 4D). Although about 4-fold higher concentrations of MCC950 are required to inhibit the NLRP3 inflammasome in U937 macrophages, cell viability was not affected by MCC950 (Fig. S5).

### Ap4-induced IL-1β release is independent of caspases, serine, cysteine, and aspartate proteases

**Fig. 5:**
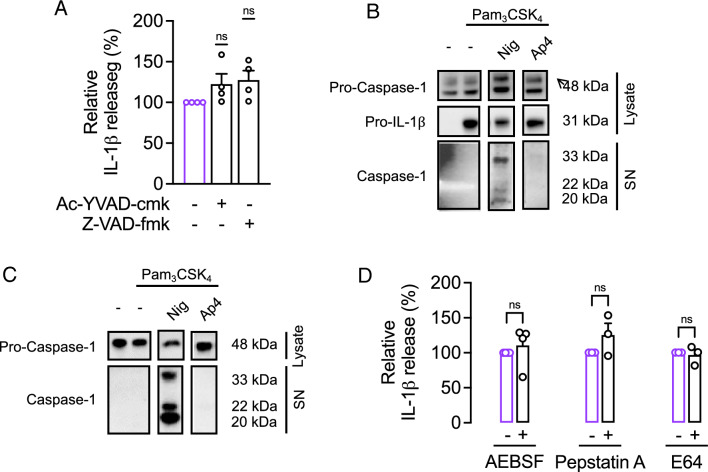
Ap4-induced IL-1β release is independent of caspases, serine, cysteine and aspartate proteases. **A** and **B** THP-1 macrophages were primed with Pam_3_CSK_4_ (1 µg/ml) for 3 h and then stimulated with Ap4 (5 mM) or **B** nigericin (10 µM) for 3 h. **A** Caspase-1 inhibitor Ac-YVAD-cmk (40 µM) or pan-caspase inhibitor Z-VAD-fmk (40 µM) was added 1 h before stimulation. IL-1β release in cell culture supernatants was determined by ELISA. Ap4-induced IL-1β release was set to 100%. All other values were calculated accordingly. Mean ± SEM (*n* = 4). One-sample *t*-test against 100%, ns ≥ 0.05. **B** Pro-caspase-1 and pro-IL-1β protein levels from cell lysates and caspase-1 protein levels from cell culture supernatants (SN) were determined by western blot analysis. The data represent two independent experiments. **C** THP-1 monocytes were primed with Pam_3_CSK_4_ (1 µg/ml) for 3 h and then stimulated with Ap4 (5 mM) or nigericin (10 µM) for 6 h. Pro-caspase-1 protein levels from cell lysates and caspase-1 protein levels from cell culture supernatants (SN) were determined by western blot analysis. The data represent two independent experiments. **D** THP-1 macrophages were primed with Pam_3_CSK_4_ (1 µg/ml) for 3 h and then stimulated with Ap4 (5 mM) for 3 h. Inhibitors of serine protease (AEBSF, 300 µM), cysteine protease (E64, 10 µM) or aspartate protease (pepstatin A, 50 µM) were added 1 h before stimulation. IL-1β release in cell culture supernatants was determined by ELISA. Ap4-induced IL-1β release was set to 100%. All other values were calculated accordingly. Mean ± SEM (*n* = 3–4). One-sample *t*-test against 100%, Statistical differences: ns ≥ 0.05

Since Ap4 activates a signaling pathway leading to NLRP3-independent IL-1β release, we aimed to determine whether caspase-1 is involved in IL-1β processing by Ap4 in the absence of NLRP3 inflammasome activity. Previously, we showed that the caspase-1 inhibitor Ac-YVAD-cmk and the pan-caspase inhibitor Z-VAD-fmk inhibit NLRP3-dependent IL-1β release (Bockstiegel et al. 2023). Inhibition of caspase activity failed to reduce Ap4-induced IL-1β secretion (Fig. 5A) indicating that caspase-1 is not involved in the cleavage of pro-IL-1β to the active form in Ap4-stimulated macrophages. This was confirmed by Western blot analysis. As caspase-1 can cleave and thus activate itself at different sites, different compositions for active caspase-1 have already been demonstrated (Boucher et al. 2018). In most cell stimulation studies, cell lysates or cell-free supernatants from stimulated immune cells are analyzed for the cleavage fragments p10 and/or p20 (Broz et al. 2010; Gritsenko et al. 2020). However, other studies have also identified p33 species as active forms of caspase-1 (Boucher et al. 2018). While the p33 form of activated caspase-1 dominated in the supernatant of Pam_3_CSK_4_-primed THP-1 macrophages stimulated with nigericin (Fig. 5B), both the p33 and p20 forms were detected in THP-1 monocytes (Fig. 5C). In contrast, no cleavage of pro-caspase-1 occurred in Ap4-stimulated primed macrophages and monocytes.

A number of other proteases have been implicated in NLRP3- and caspase-1-independent processing of pro-IL-1β (Afonina et al. 2015). However, Ap4-induced IL-1β release was not affected by either the serine protease inhibitor AEBSF or the cysteine protease inhibitor E64 (Fig. 5D). Cytokine levels also remained unchanged in the presence of the aspartate protease inhibitor pepstatin A. These results suggest that Ap4 activates a signaling pathway that results in IL-1β release, which is also independent of serine, cysteine, or aspartate proteases.

### Ap4 induces expression of metallothioneins

**Fig. 6:**
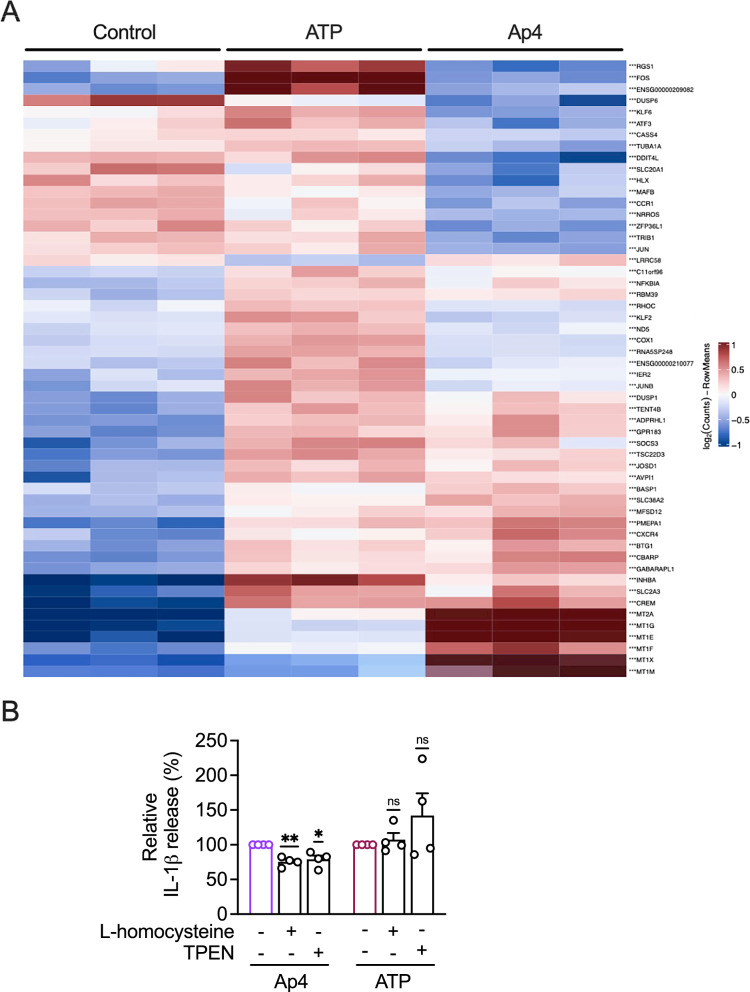
Differential gene expression and metal ion regulation in ATP- and Ap4-mediated IL-1β release. **A** THP-1 macrophages were stimulated for 3 h without or with Ap4 (5 mM) or ATP (5 mM). Afterwards RNA sequencing was performed. Gene expression data for 54 differentially expressed genes (DEGs) is heat mapped by intensity of colour as log2 fold change from three independent experiments, with red representing higher expression and blue representing lower expression. Log2 (normalized counts per million) are scaled by row. DEGs defined as genes with a log two-fold change value of less than − 1 or more than 1. **B** THP-1 macrophages were primed with Pam_3_CSK_4_ (1 µg/ml) for 3 h. l-homocysteine (100 µM) or ion chelator TPEN (10 µM) were added after priming for 1 h. Afterwards, cells were stimulated with Ap4 (5 mM) or ATP (5 mM). IL-1β release in cell culture supernatants was determined by ELISA. IL-1β release induced by Ap4 was set to 100%. All other values were calculated accordingly. Mean ± SEM (*n* = 3). One-sample *t*-test against 100%, ns ≥ 0.05, *P ≤ 0.05, **P ≤ 0.01

Our findings suggest that ATP and Ap4 regulate IL-1β release through different mechanisms. Next, we used RNAsequencing to characterize and compare the genome-wide expression profiles of ATP and Ap4 in macrophages. Compared to ATP and control, Ap4 increased the expression of metallothioneins (MTs) *MT2A*, *MT1G*, *MT1E*, *MT1F*, *MT1X*, and *MT1M* (Fig. 6A), which can be associated with inflammatory reactions (Dai et al. 2021; Wang et al. 2023). While ATP increases the expression of *FOS *(Fos proto-oncogene, AP-1 transcription factor subunit), which can be induced by P2X7 receptor agonists (Gavala et al. 2008), the expression levels remain unchanged by Ap4 stimulation. The volcanoplot shows that Ap4 causes significant changes in the expression of several genes, with genes being both up- and down-regulated (Fig. S6A). ATP, on the other hand, causes increased upregulation of several genes. Gene ontology (GO) enrichment analysis showed that by both ATP and Ap4 in THP-1 macrophages, the most altered biological processes are ‘’stress responses to metal ions’’, ‘’cellular response to zinc ions’’ and ‘’cellular response to copper’’ (Fig. S6B), which at least in the case of Ap4 could be related to the increased expression of MTs, as these bind metal ions such as zinc and copper (Carpenè et al. 2007).

To explore the role of metal ions in Ap4-stimulated cells, we tested whether l-homocysteine, which targets MTs (Barbato et al. 2007), or the membrane-permeant zinc-specific chelator TPEN (Brough et al. 2009; Cao et al. 2001) influenced the production of IL-1β. l-homocysteine and TPEN reduced the release of IL-1β by Ap4 to 75 ± 3% and 79 ± 5%, respectively (Fig. 6B). In contrast, ATP-induced IL-1β release was neither influenced by l-homocysteine nor TPEN.

## Discussion

Ap4 is a naturally occurring nucleotide known for its role in mediating vasoconstrictor effects (Tölle et al. 2008; Gómez-Villafuertes et al. 2000). Other nucleotides, such as ATP, and dinucleotides, such as uridine adenosine tetraphosphate, play critical roles in modulating cardiovascular effects and immune responses by acting as pro-inflammatory agents (Idzko et al. 2014; Schuchardt et al. (2011); Zhou et al. 2019). The current study presents the first evidence of the involvement of Ap4 in inflammatory reactions. Specifically, we found that Ap4 induced bioactive IL-1β release from primed immune cells. Modulatory effects on other cytokines were not observed indicating that IL-1β plays a central role in the Ap4-mediated inflammatory response.

Activation of the P2X7 receptor by ATP triggers IL-1β release (Di Virgilio et al. 2017; Bockstiegel et al. 2023). Due to its structural similarity to ATP, we examined whether Ap4 could also trigger IL-1β release via the P2X7 receptor. Interestingly, IL-1β release induced by ATP and Ap4 is differently modulated by the P2X7 receptor. In human macrophages, ATP-induced release of IL-1β is inhibited by the P2X7 receptor antagonist A804598 and partially by the P2 receptor antagonist PPADS (Bockstiegel et al. 2023). In contrast, both antagonists showed no effect on Ap4-induced IL-1β release. Conversely, oxATP reduced both ATP- (Bockstiegel et al. 2023) and Ap4-mediated IL-1β levels. Although oxATP is recognized as an irreversible P2X7 receptor antagonist, off-target effects have been described (Beigi et al. 2003).

To gain deeper insight into the molecular mechanism of Ap4, we investigated P2X7 receptor-mediated calcium influx and membrane pore formation in transfected HEK293 cells. BzATP but not Ap4 triggered P2X7 receptor-dependent calcium influx and pore formation indicating that Ap4 does not mediate any effect via the P2X7 receptor. However, P2X7 receptor-induced IL-1β release was reduced by BzATP in the presence of Ap4. It is hypothesized that ATP analogues like BzATP bind to the ATP binding site on the P2X7 receptor (Jiang et al. 2021). Therefore, while Ap4 might interact with the ATP binding site on the P2X7 receptor, it might fail to fully activate the receptor, leading to the absence of P2X7-dependent pore formation and ion flux, such as calcium influx and potassium current. Additionally, steric hindrance by Ap4 could impede ion flux. In the future, binding studies of Ap4 to the human P2X7 receptor could provide valuable insights into its role in Ap4-induced IL-1β release. While the P2X7 receptor among the purinergic receptors is primarily involved in the release of IL-1β, there is evidence that the P2X4 receptor also contributes to this process (Kanellopoulos et al. 2021). ATP and Ap4 both activate P2X4 receptors (Jones et al. 2000). However, IL-1β release induced by Ap4 is independent of the P2X4 receptor, consistent with previous observations regarding ATP (Bockstiegel et al. 2023).

In THP-1 cells, we found that low ATP and Ap4 concentrations trigger calcium influx. Ap4 induced significantly less calcium influx in THP-1 macrophages compared to ATP, whereas Ap4-induced calcium influx in THP-1 monocytes mirrors that induced by ATP. These findings align with studies in rat synaptosomes, where Ap4 and ATP trigger similar calcium influx, although Ap4 is more potent in human macrophages (EC_50_ = 4.0 µM) than in rat synaptosomes (EC_50_= 19.6 µM) (Gómez-Villafuertes et al. 2000). The underlying factors for the differences in ATP-mediated calcium influx between THP-1 monocytes and macrophages remain unexplored, but it has been suggested that varying expression of P2Y receptors may play a role (Layhadi and Fountain 2017).

Several studies indicate the involvement of different ionotropic P2X receptors in Ap4-mediated responses (Tölle et al. 2008; Gómez-Villafuertes et al. 2000; Jones et al. 2000; Pintor et al. 2004). While THP-1 monocytes express P2X receptors such as P2X1, P2X4 and P2X7 receptors (Layhadi and Fountain 2017), our results suggest that Ap4-induced calcium influx in THP-1 monocytes is independent of P2X receptor activity. Ap4-mediated calcium response in monocytes could not be reduced by PPADS. The different responses of THP-1 monocytes and macrophages to Ap4, despite similar expression levels of P2X receptors (Layhadi and Fountain 2017), support the assumption that Ap4-induced calcium influx functions independently of P2X receptors. Instead, our results suggest that Ap4 triggers calcium influx in THP-1 monocytes via activation of GPCRs coupled to G_q/11_, which include P2Y1, P2Y2, P2Y4 and P2Y6 receptors (Von Kügelgen and Hoffmann 2016). This conclusion is supported by previous findings showing that Ap4 activates endothelial P2Y1 receptors (Westhoff et al. 2003).

It has previously been hypothesized that nucleotides may influence the NLRP3 inflammasome via purinergic receptors other than the P2X7 receptor, leading to IL-1β release (Gombault et al. 2013). In particular, P2Y receptors have been associated with NLRP3 activation and IL-1β release in mice (Zhang et al. 2020), yet this may not be transferred to human cells (Wissemann et al. 2023). Although the Ap4-induced calcium influx could be completely blocked by YM-254,890, the G_q/11_ inhibitor had no effect on Ap4-induced IL-1β release in human macrophages, suggesting that P2Y receptors are not involved in Ap4-induced IL-1β release.

IL-1β is implicated with various inflammatory diseases, and the role of the NLRP3-caspase-1 axis in regulating IL-1β release has been extensively studied in recent years. In contrast, the significance of NLRP3- and caspase-1-independent IL-1β formation remains less investigated. We and others have demonstrated that IL-1β release induced by ATP in human macrophages is partially NLRP3-independent, and IL-1β induction by the P2X7 receptor operates independently of the NLRP3 inflammasome (Bockstiegel et al. 2023; Coll et al. 2015). Here, we demonstrated that both pharmacological and genetic inhibition of NLRP3 fails to modulate Ap4-mediated IL-1β release. Potassium efflux across the plasma membrane is a well-established mechanism contributing to NLRP3 inflammasome activation (Pétrilli et al. 2007; Muñoz-Planillo et al. 2013). Consistent with the observation that Ap4-induced IL-1β release occurs independently of NLRP3, we found that Ap4-induced IL-1β release is also unaffected by potassium efflux. It should be noted that mechanisms independent of potassium have been identified for IL-1β release; however, these mechanisms still require NLRP3 inflammasome activation (Groß et al. 2016). Additionally, Ap4 failed to trigger caspase-1 activation and neither inhibition of caspase-1 nor pan-caspases reduced Ap4-induced IL-1β release. These findings suggest that the processing of pro-IL-1β functions entirely independently of caspases, unlike ATP-induced IL-1β release (Bockstiegel et al. 2023). Serine, aspartic, and cysteine proteases are not involved in the proteolytic processing of IL-1β by Ap4. IL-1β release in macrophages requires membrane permeability (He et al. 2015; Martín-Sánchez et al. 2016). Ap4 induced membrane permeability, as evidenced by increased LDH levels. Importantly, this effect was observed irrespective of priming, suggesting that membrane permeability occurs independently of the secondary activation signal. These findings align with previous evidence suggesting that the second signal of NLRP3 inflammasome activation acts as a cytotoxic signal, promoting IL-1β release (Cullen et al. 2015). In PBMCs, Ap4 triggered IL-1β release without inducing LDH release, suggesting that IL-1β secretion occurs via an alternative pathway independent of pyroptosis (Gaidt et al. 2016).

To further delineate the molecular mechanisms of Ap4-mediated IL-1β release, we performed gene expression profiling and observed an upregulation in the expression of MT1 and MT2 genes following exposure to Ap4. While MTs have recently gained recognition as regulators of immune and inflammatory responses, the precise mechanisms of action remain unclear (Dai et al. 2021). MTs are rich in cysteine residues that coordinate multiple zinc and copper atoms under physiological conditions which underlines their key role in metal metabolism. Our findings indicate that both MTs and zinc are at least partially involved in Ap4-induced IL-1β release but are dispensable for ATP. Further studies are needed to better define the functional role of MTs in the regulation of inflammatory signaling and most notably in the specific release of IL-1β.

## Conclusions

In summary, our findings suggest the existence of a previously unrecognized pathway for the release of IL-1β by Ap4 that is NLRP3- and caspase-1-independent but involves MTs and zinc. By identifying Ap4 as a danger signal for IL-1β release, this study enhances our understanding of “inflammaging” as a driver of cardiovascular disease.

## Supplementary Information

Supplementary material 1.

## Data Availability

All data generated and analyzed during this study are included in the manuscript. The RNA sequencing data discussed in this publication have been deposited in NCBI’s Gene Expression Omnibus (Edgar 2002) and are accessible through GEO Series accession number GSE272546 (https://www.ncbi.nlm.nih.gov/geo/query/acc.cgi?acc=GSE272546).

## Abbreviations

Ap4: Adenosine 5′-tetraphosphate
ATP: Adenosine triphosphate
DMEM: Dulbecco’s modified Eagle’s medium
DEG: Differentially expressed gene
DMSO: Dimethyl sulfoxide
ELISA: Enzyme-linked immunosorbent assay
FBS: Fetal bovine serum
HBSS: Hank’s Balanced Salt Solution
IL: Interleukin
LDH: Lactate dehydrogenase
LPS: Lipopolysaccharide
MT: Metallothionein
NLRP3: Nucleotide-binding oligomerization domain, leucine-rich repeat receptor, and pyrin-domain containing-protein 3
PBMCs: Peripheral blood mononuclear cells
PBS: Phosphate buffered saline
PEI: Polyethylenimine
PMA: Phorbol 12-myristate 13-acetate
RFU: Relative fluorescence unit
TLR: Toll-like receptor
TNF: Tumor necrosis factor
