# Supplementary material for: The vasoconstrictor adenosine 5′-tetraphosphate is a danger signal that induces IL-1β
Source: Mol Med. 2025 Feb 21;31:72. doi: 10.1186/s10020-025-01116-6 (PMC11844157; doi:10.1186/s10020-025-01116-6)
Supplement: Supplementary file 1 — Supplementary material 1. [file 10020_2025_1116_MOESM1_ESM.pdf]

Supplementary Information

The vasoconstrictor adenosine 5'-tetraphosphate is a danger signal that induces IL-1β

Judith Bockstiegel[1], Jonas Engelhardt[1], Mirjam Schuchardt[2,3], Markus Tölle[2],

Günther Weindl[1*]

[1] Pharmaceutical Institute, Pharmacology and Toxicology section, University of Bonn, Gerhard-Domagk-Str. 3, 53121 Bonn, Germany.

[2] Charité-Universitätsmedizin Berlin, Corporate Member of Freie Universität Berlin and Humboldt Universität zu Berlin, Department of Nephrology and Medical Intensive Care, Hindenburgdamm 30, 12203 Berlin, Germany.

[3] Medical School Berlin, Faculty of Medicine, Rüdesheimer Straße 50, 14513 Berlin, Germany.

* Corresponding author: Dr. Günther Weindl, Pharmacology and Toxicology section, Pharmaceutical Institute, University of Bonn, 53121 Bonn, Germany. Phone: +49 228 739103, E-mail address: guenther.weindl@uni-bonn.de, ORCID: 0000-0002-4493-7597

This PDF file includes:

Supplementary Figures 1 to 7

Uncropped images of Western blots in Supplementary Figure 7

Supplementary Figures

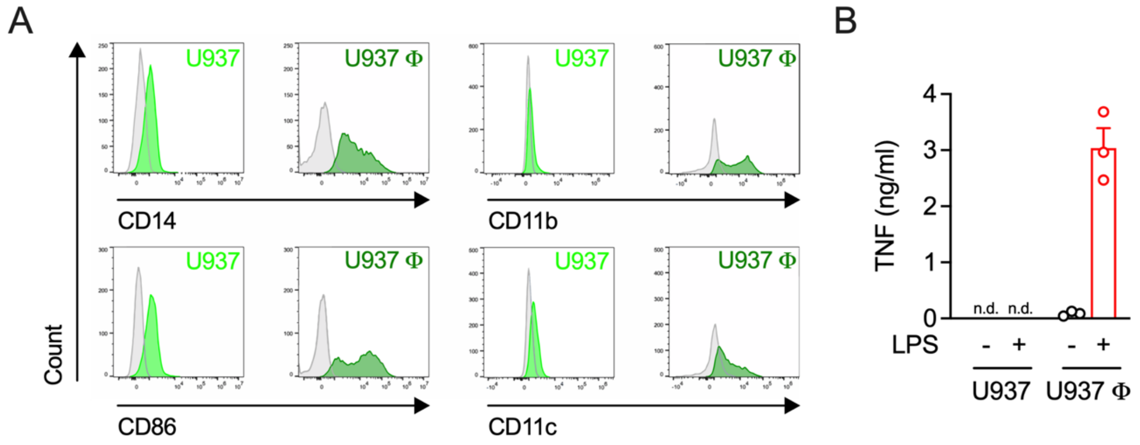

Fig. S1. Macrophages generated from U937 promonocytes express typical differentiation markers and respond to LPS. (A) U937 cells were stimulated for 72 h without or with 50 ng/ml PMA. This was followed by a 24-hour recovery period in PMA-free medium. Flow cytometry was used to measure the change in surface markers CD14, CD11b, CD11c and CD86 (coloured curves) compared to the respective fluorescence minus one (FMO) controls (grey curves). The graphs are representative of two independent experiments. Following the recovery phase, cells were stimulated with LPS *E. coli* (10 ng/ml). TNF secretion in the cell culture supernatants was determined after 4 h by ELISA. Mean ± SEM (n = 3).

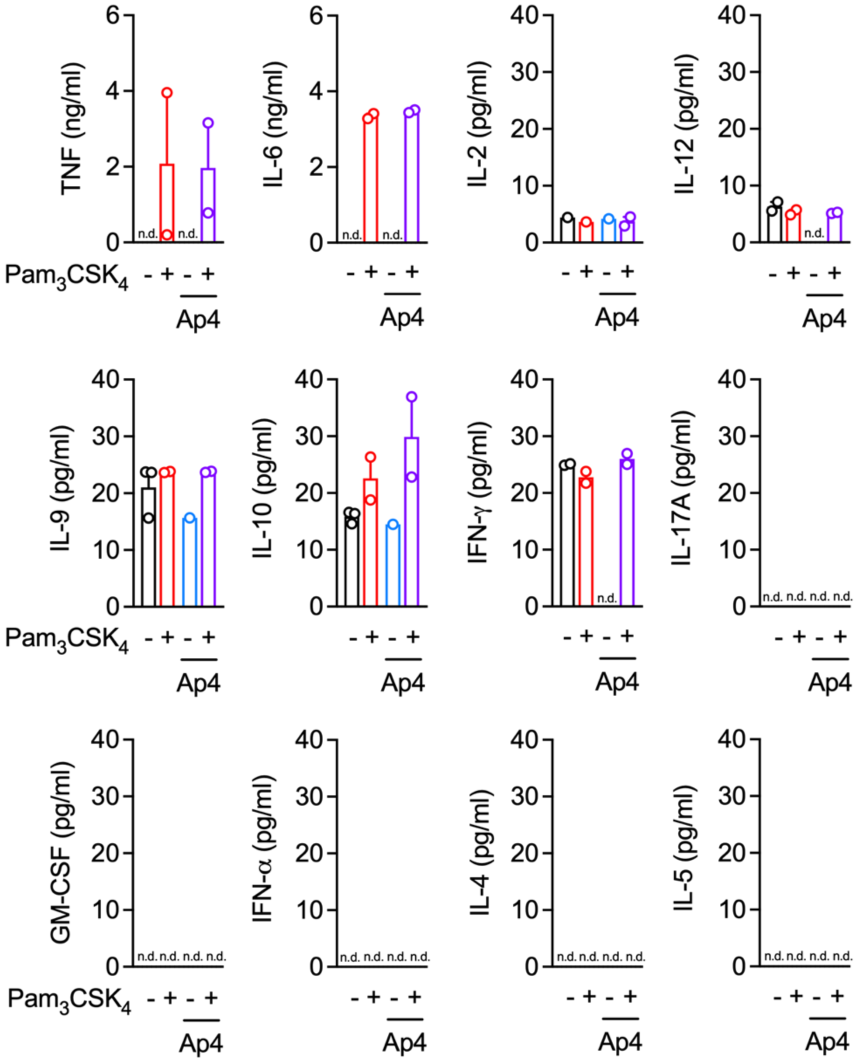

Fig. S2. Ap4 does not modulate cytokine release in unprimed and primed PBMCs. PBMCs were primed without or with Pam_3CSK_4 (1 µg/ml) for 3 h and then stimulated with Ap4 (5 mM) for 3 h. Cell culture supernatants were analyzed for cytokine concentrations by multiplex assay using flow cytometry. n.d. = not detected. Mean ± SEM (n = 2).

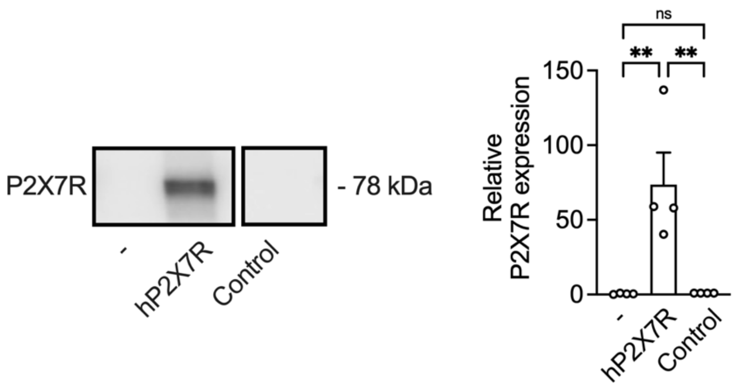

Fig. S3. Transfection of HEK293 cells with P2X7 receptor. HEK293 cells were transfected with the hP2X7 receptor (pUNO1-hP2RX7) or the control plasmid (pUNO-mcs). P2X7 receptor protein levels from cell lysates of transfected and untransfected HEK293 cells were determined by Western blot analysis. The data represent four independent experiments. P2X7 receptor protein levels were normalized to the respective total protein levels and the respective values were referred to the control (control denoted by the value 1). Mean ± SEM (n = 4). One-way ANOVA followed by Tukey´s post-test, ns ≥ 0.05, **P ≤ 0.01.

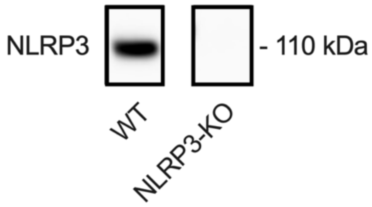

Fig. S4. NLRP3 protein levels of THP-1 macrophages and NLRP3-KO THP-1 macrophages. NLRP3 protein levels from cell lysates of THP-1 macrophages (WT) and NLRP3-KO THP-1 macrophages (NLRP3-KO) were determined by Western blot analysis. The data represent four independent experiments.

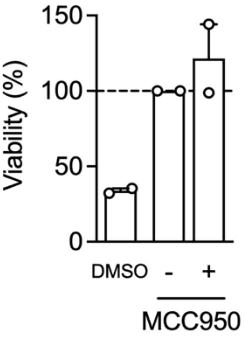

Fig. S5. Cell viability of U937 macrophages is not affected by MCC950. U937 macrophages were stimulated with MCC950 (40 µM). Cell viability was determined after 7 h by MTT assay, using DMSO (10%, v/v) as cytotoxic control. Viability was referred to the unstimulated control (control set to 100%). Mean ± SEM (n = 2).

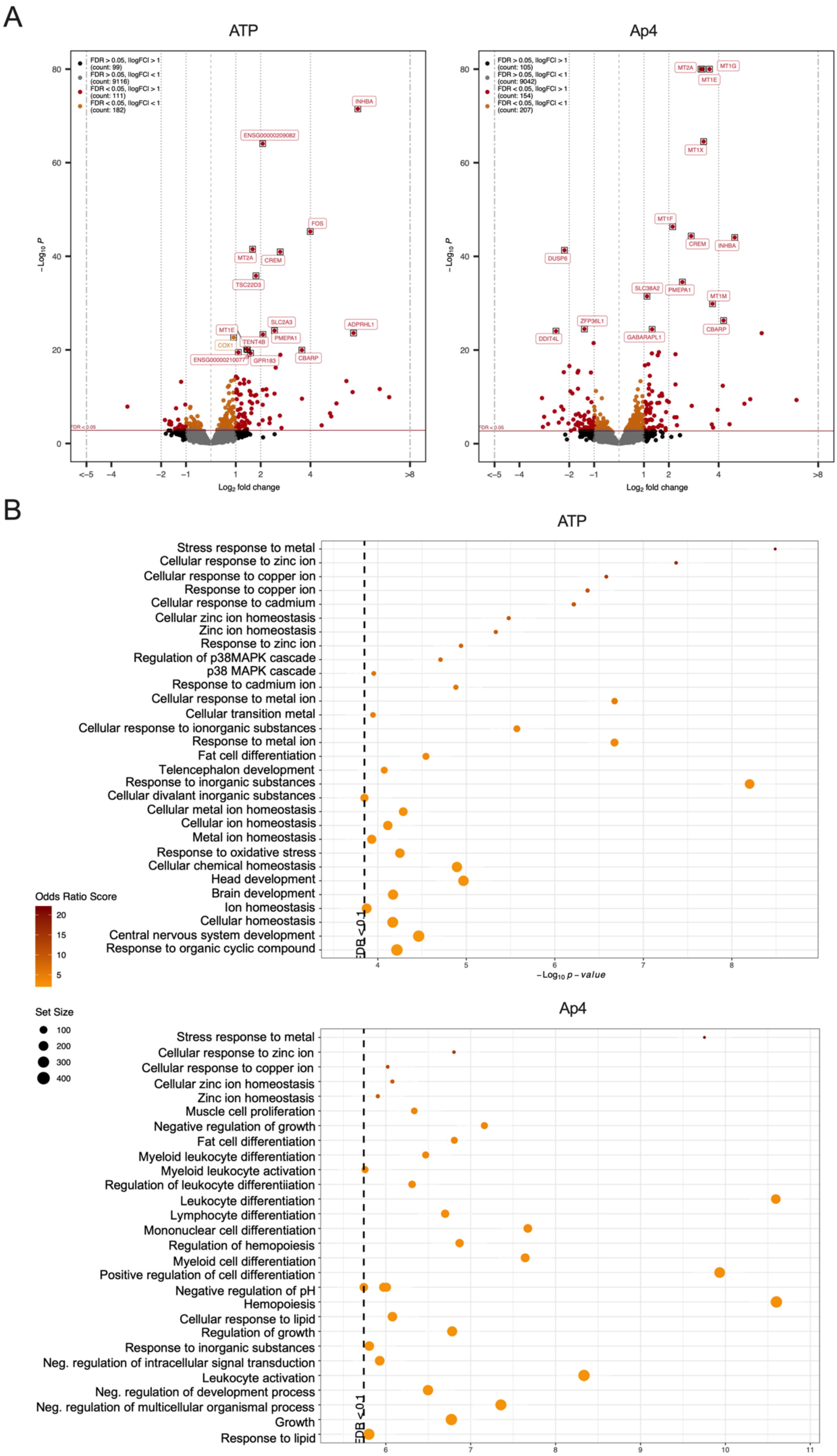
A
ATP
Ap4
FDR > 0.05, |logFC| > 1 (count: 99)
FDR > 0.05, |logFC| < 1 (count: 9116)
FDR < 0.05, |logFC| > 1 (count: 111)
FDR < 0.05, |logFC| < 1 (count: 182)
FDR > 0.05, |logFC| > 1 (count: 105)
FDR > 0.05, |logFC| < 1 (count: 9042)
FDR < 0.05, |logFC| > 1 (count: 154)
FDR < 0.05, |logFC| < 1 (count: 207)
INHBA
ENSG00000209082
FOS
MT2A
CREM
TSC22D3
SLC2A3
ADPRHL1
MT1E
TENT4B
PMEPA1
COX1
ENSG00000210077
GPR183
CBARP
MT1G
MT1X
MT1F
DUSP6
SLC38A2
MT1M
ZFP36L1
DDIT4L
GABARAPL1
FDR < 0.05
−Log10 P
Log2 fold change
B
Stress response to metal
Cellular response to zinc ion
Cellular response to copper ion
Response to copper ion
Cellular response to cadmium
Cellular zinc ion homeostasis
Zinc ion homeostasis
Response to zinc ion
Regulation of p38MAPK cascade
p38 MAPK cascade
Response to cadmium ion
Cellular response to metal ion
Cellular transition metal
Cellular response to ionorganic substances
Response to metal ion
Fat cell differentiation
Telencephalon development
Response to inorganic substances
Cellular divalant inorganic substances
Cellular metal ion homeostasis
Cellular ion homeostasis
Metal ion homeostasis
Response to oxidative stress
Cellular chemical homeostasis
Head development
Brain development
Ion homeostasis
Cellular homeostasis
Central nervous system development
Response to organic cyclic compound
FDR <0.1
−Log10 p − value
Odds Ratio Score
Set Size
Stress response to metal
Cellular response to zinc ion
Cellular response to copper ion
Cellular zinc ion homeostasis
Zinc ion homeostasis
Muscle cell proliferation
Negative regulation of growth
Fat cell differentiation
Myeloid leukocyte differentiation
Myeloid leukocyte activation
Regulation of leukocyte differentiiation
Leukocyte differentiation
Lymphocyte differentiation
Mononuclear cell differentiation
Regulation of hemopoiesis
Myeloid cell differentiation
Positive regulation of cell differentiation
Negative regulation of pH
Hemopoiesis
Cellular response to lipid
Regulation of growth
Response to inorganic substances
Neg. regulation of intracellular signal transduction
Leukocyte activation
Neg. regulation of development process
Neg. regulation of multicellular organismal process
Growth
Response to lipid

Fig. S6. ATP and Ap4 differentially regulate gene expression. THP-1 macrophages were stimulated for 3 h without or with Ap4 (5 mM) or ATP (5 mM). Afterwards RNA sequencing was performed. (A) Volcano plot of all 9508 identified genes indicate differentially expressed genes after treatment with ATP (left) or Ap4 (right) compared to control (RPMI). The negative $\log_{10}$ transformed p-values (y-axis) were plotted against the average $\log_2$-fold change (x-axis) in gene expression. Transcripts with a p-value < 0.5 were classified as statistically significant (red lines indicate cut-off values for p-value). Differentially expressed gene (DEGs) defined as genes with a log two-fold change value of less than -1 or more than 1. Significantly differentially expressed transcripts are denoted in red. (B) Dot plot of gene ontology (GO) enrichment analysis showing enriched biological processes, sorted by absolute score. Colors indicate the odds ratio and dots size represent the number of DEG in the given pathway.

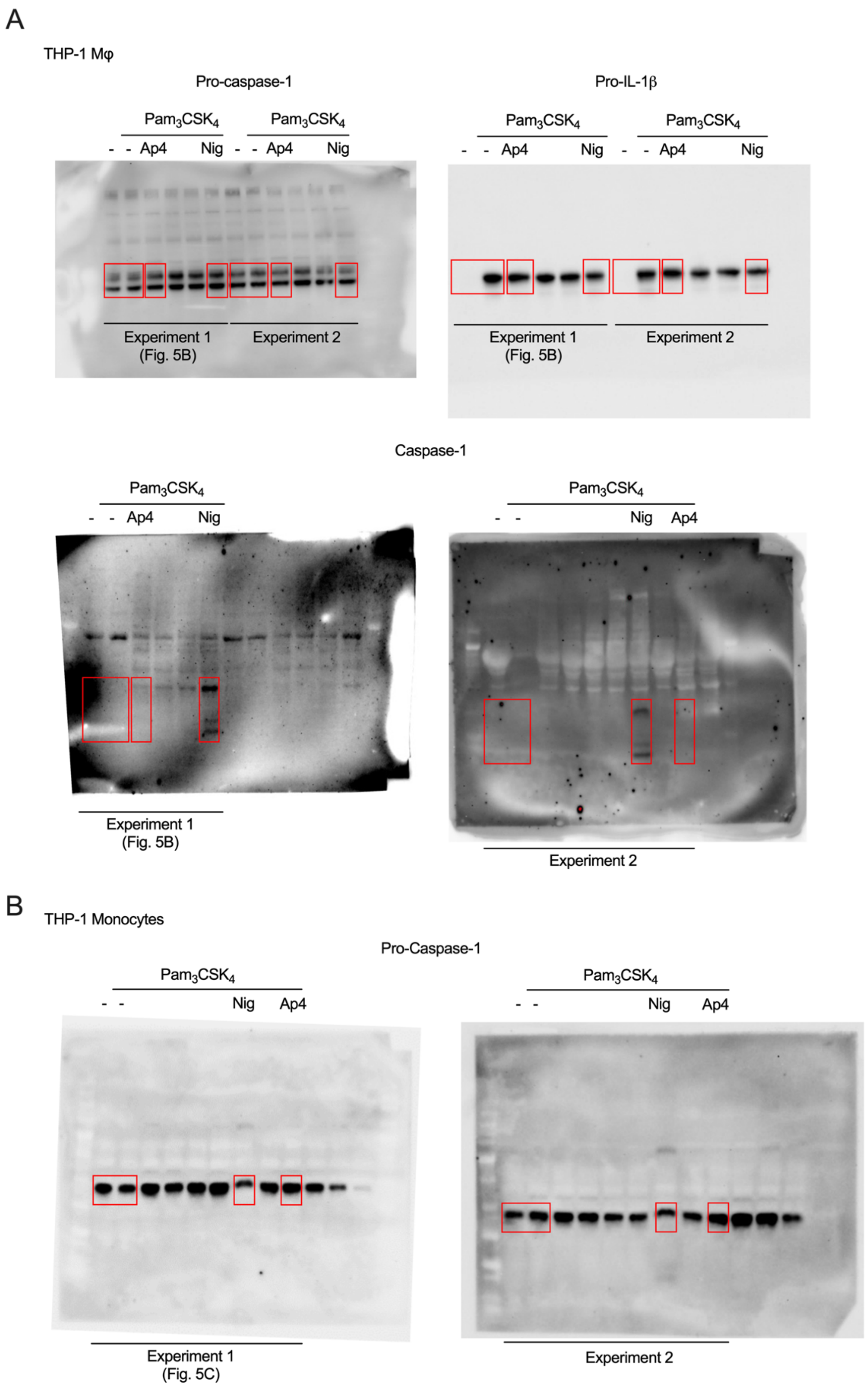
A
THP-1 Mφ
Pro-caspase-1
Pam_3CSK_4 Pam_3CSK_4
- - Ap4 Nig - - Ap4 Nig
Experiment 1
(Fig. 5B)
Experiment 2
Pro-IL-1β
Pam_3CSK_4 Pam_3CSK_4
- - Ap4 Nig - - Ap4 Nig
Experiment 1
(Fig. 5B)
Experiment 2
Caspase-1
Pam_3CSK_4
- - Ap4 Nig
Experiment 1
(Fig. 5B)
Pam_3CSK_4
- - Nig Ap4
Experiment 2
B
THP-1 Monocytes
Pro-Caspase-1
Pam_3CSK_4
- - Nig Ap4
Experiment 1
(Fig. 5C)
Pam_3CSK_4
- - Nig Ap4
Experiment 2

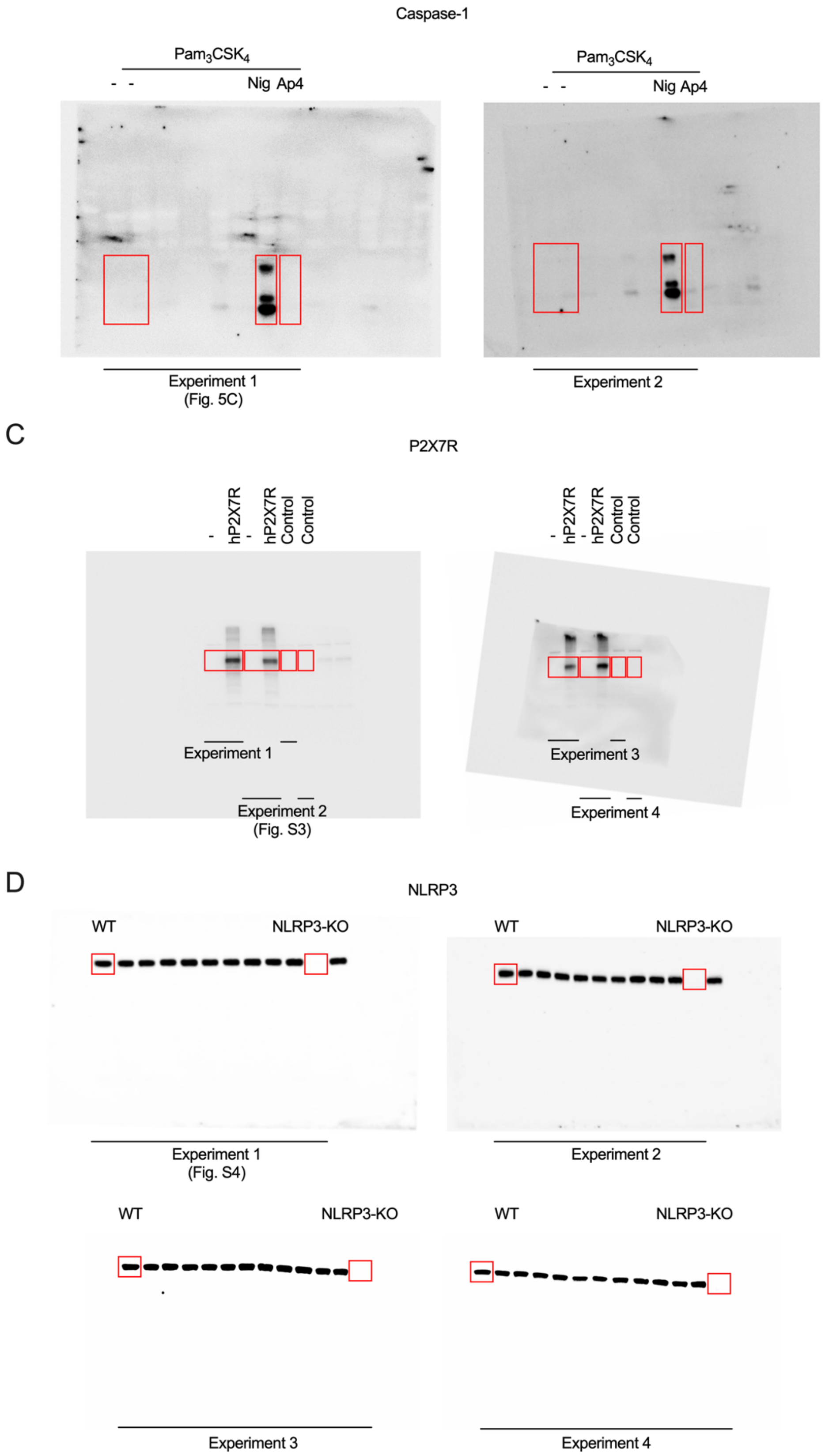

Fig. S7. Uncropped images of the original western blots. (A) Complete western blots of pro-caspase-1, pro-IL-1β and caspase-1 shown in Fig. 5B. (B) Complete western blots of pro-caspase-1 and caspase-1 shown in Fig. 5C. (C) Complete western blots of P2X7R shown in Fig. S3. (D) Complete western blots of NLRP3 shown in Fig. S4.
